# The relationship of major depressive disorder with Crohn's disease activity

**DOI:** 10.1016/j.clinsp.2023.100188

**Published:** 2023-03-28

**Authors:** Carolina Bortolozzo Graciolli Facanali, Carlos Walter Sobrado Junior, Renério Fraguas Junior, Marcio Roberto Facanali Junior, Lucas Rodrigues Boarini, Lucas Faraco Sobrado, Ivan Cecconello

**Affiliations:** a Colorectal Surgery Division, Department of Gastroenterology, Hospital das Clínicas, Faculdade de Medicina da Universidade de São Paulo, São Paulo, SP, Brazil; b Department and Institute of Psychiatry, Hospital das Clínicas, Faculdade de Medicina da Universidade de São Paulo, São Paulo, SP, Brazil; c Divisão de Psiquiatria e Psicologia no Hospital Universitário da Universidade de São Paulo, São Paulo, SP, Brazil

**Keywords:** Crohn's disease, Depression, Patient Health Questionnaire, Phenotype, Inflammatory bowel disease

## Abstract

**Introduction:**

Crohn's disease (CD) has been related to an increased prevalence of psychiatric disorders and suicide risk (SR). However, the nature of their relationship still deserves clarification. The aim of this study is to assess the prevalence of major depressive disorder (MDD) in patients with CD, and to investigate the relationship between MDD and CD outcomes.

**Methods:**

A cross-sectional study involving CD patients was performed. CD activity was evaluated by the Harvey-Bradshaw index and CD phenotype by the Montreal classification. The presence of MDD was assessed by the Patient Health Questionnaire score-9 (PHQ-9). Sociodemographic data and other characteristics were retrieved from electronic medical records.

**Results:**

283 patients with CD were included. The prevalence of MDD was 41.7%. Females had a risk of MDD 5.3 times greater than males. CD disease duration was inversely correlated with MDD severity. Individuals with active CD were more likely to have MDD (OR = 796.0; 95% CI 133.7‒4738.8) than individuals with CD remission. MDD was more prevalent in inflammatory behavior (45.5%) and there were no statistical differences regarding the disease location. 19.8% of the sample scored positive for SR.

**Conclusion:**

The present results support data showing an increased prevalence of MDD in individuals with CD. Additionally, it indicates that MDD in CD might be related to the activity of CD. Prospective studies are warranted to confirm these results and to address whether MDD leads to CD activity, CD activity leads to MDD or both ways are existent.

## Highlights

• There is a high prevalence of depressive symptoms in patients with Crohn's disease.
• Depressive symptoms are related to the active disease.
• According to the Montreal classification, the penetraing behavior of Crohn's disease, was less related a rates of depression than inflammatory behavior disease.

## Introduction

The World Health Organization (WHO) estimates that Major Depressive Disorder (MDD) can affect 3% to 11% of the global population, with a higher prevalence in women than in men.^1^ MDD might be severe, with a persistent or recurrent episodic course. It is one of the most important health problems in the world.

The progression of Inflammatory Bowel Disease (IBD) with worsening diarrhea and bleeding has been related to an increase in the prevalence and severity of depression.^2^ The negative impact of depression or depressive symptoms has been supported for both, CD and UC.^3^^,^^4^ Also, previous diagnosis of depression has been associated with a higher incidence of CD and UC over time.^5^ However, depressive symptoms have been associated with clinical recurrence over time more intensely in patients with CD than in Ulcerative Colitis (UC).

Considering specifically Crohn's Disease (CD), a systematic review of 158,371 adult individuals, found a pooled prevalence of depressive symptoms of 25.3%.^6^ However, no consensus on the prevalence of depression in individuals with CD does exist.^6^ For example, in a study, where the mean age of the patients was 42 years and the mean duration of the disease was 14 years, the prevalence of depressive symptoms was 3%, with no significant differences between patients with CD and UC.^7^ Also, studies have not found an association between depressive status and IBD severity or activity.^7^^,^^8^ In addition, there are few studies in countries where IBD is considered emerging, such as Brazil. The aim of this study is to assess the prevalence of MDD in patients with CD and to investigate the relationship between MDD and CD outcomes.

## Methods

### Study design and population

A cross-sectional study was performed at a reference center of a teaching general hospital for IBD in Brazil. All patients diagnosed with CD between September 1, 2019, and February 30, 2020, were eligible and consecutively invited to participate in the study. Those with clinical, endoscopic, and radiological diagnoses of CD, of both genders, in activity or not, and over 14 years of age, were selected. Both, asymptomatic and having a very severe CD condition could be included.

Patients with diagnostic doubts regarding CD, as well as patients who reported reading difficulties due to low visual acuity, low education, or any other related aspect, were excluded. Patients under 18 years of age who did not have their parents' consent and participation in the entire evaluation process were also excluded.

### Sociodemographic and clinical data

Initially, a questionnaire was used to obtain sociodemographic data, including age in years, gender, level of schooling, family income, marital relationship status, occupation, and religion. Other clinical features, such as surgical history, current medications and previous treatments related to CD, age at diagnosis, duration of CD disease, smoking history, and whether the disease was active or not, were obtained through data collection in electronic medical records.

### Clinical evaluation and classification criteria

The phenotypic classification of CD disease was performed at the time of clinical evaluation, according to the Montreal classification proposed by the 2005 World Gastroenterology Working Group.^9^ Disease activity was assessed by the Harvey-Bradshaw Index (HBI).^10^ An HBI score of 5 or greater indicates active disease, and remission was defined as an HBI of less than 5. The Montreal Classification and the Harvey-Bradshaw were chosen because they are the most widely used instruments in clinical research respectively to classify CD phenotype and to assess CD activity.^11^ Biochemical laboratory assessment of Hemoglobin (Hb), Hematocrit (HT), and C-Reactive Protein (CRP) values, which contribute to the diagnosis of disease activity, were obtained from electronic medical records, which are usually collected in the same week or the week before the outpatient consultation. In the absence of any exam or if performed within a period longer than 1 month, they were redone on the same day of participation in the research.

For the purpose of carrying out this work, the authors considered the upper Gastrointestinal Tract (GIT), the segment that involves the esophagus, stomach, duodenum, and jejunum.

### Assessment of depressive symptoms, diagnosis of major depressive disorder and suicide risk

There are several instruments developed for screening, monitoring, and establishing levels of severity of depression.^12^^,^^13^ In relatively large samples, self-administered questionnaires have been used to case finding of depression mainly because of their speed, ease of filling and not requiring a qualified professional.^14^ One of the instruments used to assess depression is the 9-item Patient Health Questionnaire (PHQ-9) has an accuracy of 88%^15^ and is a self-report version derived from the Primary Care Evaluation of Mental Disorders (PRIME-MD),^15^^,^^16^ which translation and validation study has already been done in Brazil.^17^ In the last decade, a large number of publications have evaluated the diagnostic accuracy and feasibility of PHQ-9 in different populations, confirming its good performance.^18^^,^^19^ Thus, to assess depressive symptoms, and the diagnosis of MDD the authors opted for PHQ-9. The PHQ-9 has 9 questions addressing depressed mood, anhedonia, problems with sleep, tiredness or lack of energy, change in appetite or weight, feelings of guilt or worthlessness, problems with concentration, feeling slow or restless, and suicidal thoughts.^20^

Answer choices are based on a frequency of days, considering how the person felt or behaved during the previous two weeks. Each symptom is evaluated according to a Likert scale, which varies from 0 to 3 points according to the frequency with which the signs and symptoms of depression occur, considering the two weeks prior to the evaluation, with 0 = “never”, 1 = “several days”, 2 = “more than half of the days” and 3 = “almost every day”. A total score is obtained by the sum of these nine items and ranges from 0 to 27. The grading of depression is based on the following cut-off points of the sum of scores obtained: 0‒4, absence of depression; 5‒9, mild depression; 10‒14, moderate depression, 15‒19, moderate-severe depression; ≥ 20, severe depression.

The authors used the cutoff score ≥ 10 to declare MDD (moderate, moderate severe, and severe depression), an index already used by other authors.^15^^,^^16^

Item 9 of the PHQ-9 was used as the index of Suicide Risk (SR).^21^ This item exclusively assesses the frequency of thoughts of death or self-harm in the two previous weeks. Results other than “never” were considered positive for SR.

Patients who presented PHQ-9 values greater than or equal to 10 were referred for specialized follow-up with a psychiatric team at the hospital.

The physician responsible for the study (C.B.G.F) monitored the completion of the questionnaire. Guidance was provided but without interfering with the completion of the questionnaire. The Portuguese version of the questionnaire was obtained at http://www.phqscreeners.com/.

### Statistical analysis

Qualitative characteristics of patients were described using absolute and relative frequencies, and quantitative characteristics were described using summary measures (mean, standard deviation, median, minimum, and maximum). The authors performed an analysis considering the presence or absence of MDD and also considering the categories moderate, moderately severe, and severe depression. The authors used chi-square tests or exact tests (Fisher's exact test or likelihood ratio test) to investigate the association of qualitative variables. To investigate the association of quantitative variables with depression the authors used Student's t-test or Mann-Whitney test. Bivariate logistic regression analysis was performed to estimate the unadjusted Odds Ratio (OR) of the association with MDD, and the multiple logistic regression model estimated adjusted values for the variables that in the bivariate tests presented levels of significantly less than 0.10 (*p* < 0.10), with all variables inserted in the model, kept in the final model (full model). The IBM-SPSS for Windows version 20.0 software was used to perform the analyses, and the Microsoft Excel 2003 software was used for data tabulation. The tests were performed with a significance level of 5%.

### Ethical aspects

The study was approved by the Ethics Committee and registered on *Plataforma Brasil* under number 36477720.5.0000.0068.

## Results

In this study, 321 patients were identified who were invited to complete the survey, 283 CD patients signed the informed consent form, satisfied the inclusion and exclusion criteria, and were included. Thirty-eight patients were excluded due to voluntary refusal, visual impairment, or illiteracy.

The sociodemographic and clinical features of the population studied are shown in Table 1. From the 283 CD patients, 58.7% (*n* = 166) were female; mean age of 45.5 (SD = ±13,8 years; range 14 to 78 years) years; most declared being married or in a stable relationship (51.6%; *n* = 146), non-smoker (87.2%; *n* = 247), Catholic religion (43.1%; *n* = 122), having completed high school (40.6%; *n* = 115), and receive up to 3 minimum wages, equivalent to US$ 613.51 monthly (77.4%; *n* = 219). Of the 283 patients participating in this study, 86% had never passed a psychiatric evaluation for depression. Patients answered the self-report questions about MDD in 7 (range 5 to 10 minutes).

**Table 1:** Sociodemographic characteristics of the included patients with Crohn's disease.

Variable	Description
	*n* = 283 (%)
Gender	
Female	166 (58.7)
Current age (years)	
Mean ± SD	45.5 ± 13.8
Median (mín.; max.)	45 (14; 78)
Schooling	
Primary	96 (33.9)
High school	115 (40.6)
University education	72 (25.4)
Monthly income	
Up to 3-times the minimum wages	219 (77.4)
Between 3 and 5 minimum wages	52 (18.3)
5 or more minimum wages	12 (4.2)
Marital status	
Single	100 (35.3)
Married/Stable union	146 (51.6)
Divorced	25 (8.8)
Widow(er)	12 (4.2)
Religion	
Catholic	122 (43.1)
Protestant	94 (33.2)
Others	67 (23.7)
Smoker	
Yes	36 (12.7)

SD, Standard Deviation.

PHQ-9 scores, Clinical and surgical data (extracted from patients’ medical records) are presented in Table 2. The first manifestation of CD occurred at the mean age of 32.6 (SD = ± 12,9 years) years and the mean duration of the disease was 13.2 (SD = ± 8,9 years) years. Most patients had at least one previous surgery due to CD (65.8%; *n* = 86). The most frequent surgeries were anal fistulectomy (25.8%; *n* = 73) followed by right colectomy (22.3%; *n* = 63), enterectomy (20.1%; *n* = 57), and total colectomy (17%; *n* = 48).

**Table 2:** Psychiatric, clinical, and surgical characteristics of the included patients with Crohn's disease.

Variable	Description
	*n* = 283 (%)
Disease Location	
Ileal	19 (6.7)
Colonic	97 (34.3)
Ileocolonic	137 (48.4)
Ileal + TGS	28 (9.9)
Ileocolonic + TGS	2 (0.7)
Disease Behavior	
Inflammatory (non-structuring/non penetrating)	66 (23.3)
Stricturing	82 (29)
Penetrating (including perianal disease)	135 (47.7)
PHQ9	
None (score 0‒4)	99 (35)
Mild (score 5‒9)	66 (23.3)
Moderate + moderately severe + severe (score ≥10)	118 (41.7)
Suicide risk	
Yes	56 (19.8)
Age at diagnosis (year)	
16 or younger	21 (7,4)
17 to 40	192 (67.8)
Over 40	70 (24.7)
Age at diagnosis (year)	
Mean + SD	32,6+- 12,9
Median (min;max)	31(10;71)
Previous surgical treatment	
Yes	186 (65.7)
Surgery	
Anorectal fistulotomy	73 (25.8)
Ileocolectomy	63 (22.3)
Enterectomy	57 (20.1)
Total colectomy	48 (17)
Correction to abdominal fistula	22 (7.8)
Left colectomy (segmental colectomy)	5 (1.8)

PHQ, Patient Health Questionnaire; TGS, Esophagus, Stomach, Duodenum, Jejunum; SD, Standard Deviation.

In agreement with the Montreal classification, of the 283 patients with CD, the most prevalent type according to anatomical location had ileocolonic involvement (48.4%; *n* = 137) followed by those limited to the colon (34.3%; *n* = 97), ileum, and upper Gastrointestinal Tract (GIT) (9.9%, *n* = 28), ileal (6.7%; *n* = 19) and with a lower incidence of ileocolonic and GIT (0.7%; *n* = 2). Conforming to the behavior of CD, penetrating was the most frequent (47.7%; *n* = 135), followed by patients with fibrostenosing behavior 29% (*n* = 82) and finally inflammatory behavior 23.3% (*n* = 66).

In accordance with HBI, most patients were in clinical remission at baseline (54.8%; *n* = 155) while 45.2% (*n* = 128) had active CD. Of these 32.2% (*n* = 91) the disease moderate and 13.1% (*n* = 37) had severe disease. Regarding medication history, 44.1% (*n* = 125) were being treated with a single drug and 45.5% (*n* = 129) were using two or three drugs. The most used drugs were infliximab in association with azathioprine (14.5%; *n* = 41), mesalazine (13%; *n* = 38), mesalazine and azathioprine (9.9%; *n* = 28), azathioprine (9.5%; *n* = 27), adalimumab (9.2%; *n* = 26) and adalimumab and azathioprine 5.7%; *n* = 16). Twenty-nine patients (10.2%) were without medication for CD.

**Figure 1:**
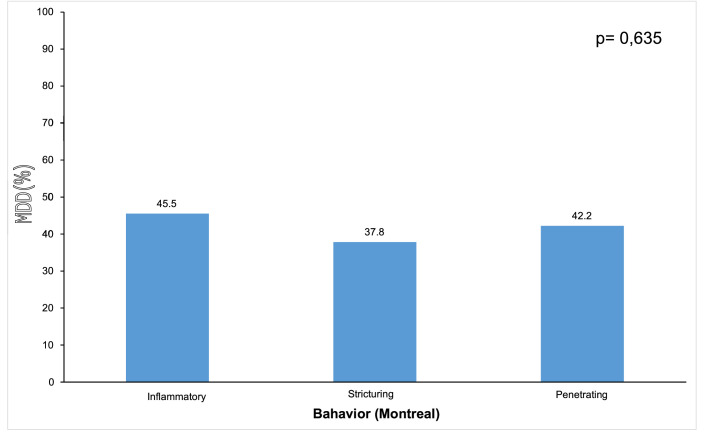
The relationship between major depressive disorder and the behavior of Crohn's disease.

Concerning the prevalence of depression according to levels of severity, based on the depression categories of the PHQ-9, among the 283 patients with CD who participated in the research, 35% (*n* = 99) had scores between 0‒4, 23.3% (*n* = 66) had scores for mild depression (score PHQ-9 5‒9) and 41.7% (*n* = 118) had moderate, moderately severe or severe depression (MDD or score PHQ- 9 ≥ 10). Of the 283 patients, 19.8% (*n* = 56) were assessed as positive for suicide ideation by item 9 of the PHQ-9. Patients classified as having MDD, (i.e., those with PHQ-9 ≥ 10) and their epidemiological and clinical features are described in Table 3. Female patients had a higher prevalence of MDD than male patients (PHQ-9 ≥ 10, *p* < 0.001). MDD was not associated with other factors such as age, level of schooling, monthly income, marital status, religion, and smoking. Of the 128 patients with CD in clinical activity, 109 (85.2%) had MDD. Of the 155 patients in clinical remission, only 9 (5.8%) had MDD (95% CI 40.54‒213.64, *p* < 0.001). Figure 1 shows the relationship between MDD and the behavior of CD.

**Table 3:** Association of major depressive disorder with sociodemographic, clinical, and surgical variables in the included patients with Crohn's disease.

Variable	MDD		OR	CI (95%)		p
	None/mild	Moderate/severe		Lower	Upper	
Gender						<0.001
Female	80 (48.2)	86 (51.8)	1.00			<0.001
Male	85 (72.6)	32 (27.4)	0.35	0.21	0.58	
Age at diagnosis (year)			1.001	0.982	1.019	0.952^a^
Mean ± SD	32.5 ± 12.9	32.6 ± 13				
Median (mín.; máx.)	31 (10; 71)	31.5 (10; 66)				
Age at onset						0.747
16 years or younger	10 (50)	11 (50)	1.00			
17 to 40 years	113 (58.9)	79 (41.1)	0.70	0.28	1.76	
40 years	40 (58)	30 (42)	0.73	0.26	1.97	
Hemoglobin (g/dL)			0.860	0.740	1.000	0.071^a^
Mean ± SD	13.2 ± 1.7	12.9 ± 1.8				
Median (mín.; máx.)	13.3 (7.6; 16.7)	12.8 (8.8; 16.8)				
Hematocrit (%)			0.930	0.880	0.980	0.006^a^
Mean ± SD	39.8 ± 4.5	38.3 ± 4.5				
Median (mín.; máx.)	40.1 (21.7; 49.3)	38 (27.5; 48.4)				
C- reactive protein (mg/dL)			1.010	1.000	1.020	0.206^b^
Mean ± SD	10 ± 17.3	15.3 ± 31.6				
Median (mín.; máx.)	3 (0.1; 97.2)	4.6 (0.3; 257)				
Surgeries						0.212
Yes	110(59.1)	76 (40.9)	1.00			
No	55 (56.7)	42 (43.3)	1.32	0.77	2.24	
Disease duration (years)			0.968	0.942	0.995	0.021^a^
Mean ± SD	14.3 ± 8.9	11,8 ± 8.6				
Median (mín.; máx.)	14 (1; 38)	11 (1; 40)				
Remission						<0.001
Yes	146 (94.2)	9 (5.8)	1.00			
No	19 (14.8)	109 (85.2)	93.06	40.54	213.64	
5-Aminosalicylic acid						0.731
Yes	61 (57)	46 (43)	1.09	0.67	1.77	
No	104 (59.1)	72 (40.9)	1.00			
Immunosuppressant						0.709
Yes	89 (59.3)	61 (40.7)	0.91	0.57	1.47	
No	76 (57.1)	57 (42.9)	1.00			
Anti-TNF biological						0.123
Yes	70 (53.4)	61 (46.6)	1.45	0.90	2.34	
No	95 (62.5)	57 (37.5)	1.00			
Corticosteroid use						>0.999*
Yes	2 (50)	2 (50)	1.41	0.20	10.12	
No	163 (58.4)	116 (41.6)	1.00			

MDD, Major Depressive Disorder; OR, Odds Ratio; CI, Confidence Interval; TNF, Tumoral Necrosis Factor; SD, Standard Deviation.

a Student *t*-test.

b Mann-Whitney test. Chi-Squared Test; # Likelihood radio test.

Variables that explained MDD according to the multiple regression analysis were female gender, HT, the activity of CD, and inflammatory behavior. Female patients were 5.3 times more likely to have MDD than male patients (95% CI 1.95‒14.48). For each 1% increase in HT, there was a 28% reduction in the chance of MDD (OR = 0.72; 95% CI 0.53‒0.97) among patients. Patients with active CD, according to HBI, were approximately 800 times more likely to have MDD (OR = 796.0; 95% CI 133.7‒4738.8) than patients in remission. Regardless of the other patients’ characteristics, the fibrostenosing and penetrating behavior presented a statistically lower chance of severe depression than the inflammatory behavior, respectively, 92% (OR = 0.08; 95% CI 0.01‒0.50) and 97% (OR = 0.03; 95% CI 0.0–0.18). Duration of CD was inversely correlated with psychiatric disorders, that is, patients with a longer diagnosis of CD were likely to be less depressed (*p* = 0.021).

## Discussion

This study consistently confirms the high prevalence of MDD among CD patients. The authors found MDD (defined as PHQ-9 ≥ 10) in 41.7% of 283 CD patients, this prevalence is in line with other studies that used the PHQ-9^22^, ^23^, ^24^, ^25^, ^26^ or other instruments for measuring depression in this population.^27^^,^^28^

Most patients who participated in the research had a mean age at diagnosis of 32.6 years, which is similar to other studies performed in Brazil^29^^,^^30^ and in other countries such as Canada,^31^ Italy,^32^ and the United States of America.^33^ At the time of this research, the mean age of the patients involved was 45.5 years, similar to other published studies.^29^^,^^34^ According to some authors, women, elderly patients, and smokers are more likely to develop depression,^35^^,^^36^ however, other authors disagree on the influence of these clinical variables as a risk factor for depression in individuals with IBD.^4^^,^^7^

This present study, formed predominantly by women (58.7%) is in agreement with an epidemiological study carried out in Brazil, involving 22,638 patients from the public health system, which showed a significant predominance of women in both CD and UC.^34^

Women were also more susceptible to MDD and were 5.32 times more likely to be at risk of depression than men (PHQ-9 ≥ 10). These results are in agreement with more recent reports where, in addition to depression, other psychological disorders such as anxiety and mood changes are more common in women than in men affected by CD.^28^^,^^32^^,^^33^

There was a more significant prevalence of MDD in patients with active disease (95% CI 40.54‒213.64; *p* < 0.001) when compared to those in remission (85.2% vs. 5.8%), making it evident that the presence of depressive symptoms was associated with disease activity, results similar to the literature, where patients with active IBD have higher rates of depression compared to those in remission.^3^^,^^6^

It was also observed in this present study that the chance of patients with disease activity having MDD is approximately 800 times greater than patients in remission, confirming that disease activity is one of the main risk factors for depression.^2^^,^^6^ Symptoms of depression have been associated with more severe symptoms of CD and are responsible for more frequent flare-ups,^37^ higher hospitalization rates,^38^ and lower adherence to treatment.^39^ In a Scandinavian study with 60 patients with IBD in remission, and 47 with CD, followed for 18 months, the authors noticed that in periods of disease exacerbation, depression scores worsened significantly and symptoms of anxiety and depression were more intense. in the first year of diagnosis,^37^ however, patients in clinical remission are not exempt from psychological suffering, in agreement with some authors.^40^ In addition, for any relapse during the period of remission of the disease, the rate of depression increases dramatically, reaching 60% and anxiety to 80%.^41^ In the present study, just over half of the patients were in remission at the beginning of the research and without symptoms of depression (*n* = 146), a fact that can be attributed to the good care and drug treatment used, formed largely by drugs that have been proven to lead to profound and sustained remission of the disease.^42^

In addition, the high incidence of depression in patients with active disease detected in this study is one of the highest rates reported in studies involving CD and may be related to the aggressiveness of the disease, since most of them had fibrostenosing and penetrating behavior. Similar reports were observed by other authors.^35^^,^^43^

On the other hand, the prevalence of depression reported in patients in remission, in the present study, is among the lowest found in the literature, possibly as a result of the patients' deep remission.^42^ These results show that the sample is well balanced, and distributed in both extremes, that is, composed of patients with active and inactive disease in adequate numbers and distribution.

A suicidal ideation rate of 19.8% (56/283 patients) was also evidenced in this study, lower than those reported in other studies where the values found exceeded 30%, however, it is important to emphasize that other tools evaluation were used.^44^^,^^45^

Also in this study, the duration of the disease was inversely correlated with depression, in other words, patients with a longer duration of illness are less susceptible to depression (p > 0.05). Probably not because these patients have not experienced all the complications that the disease causes, but perhaps because they are more resilient to emotional stress or because they are resigned to the state of the disease since the adjustment is a difficult process for a disease. chronic, repeatedly marked by periods of remissions and exacerbations.^37^

Approximately, 49.5% (*n* = 140) of the patients were using immunobiological at the time of the evaluation, and no significant differences were found in the scores for MDD between those using monotherapy or combined therapy with immunosuppressants and, also, with those who were not using biologicals. It was evident that the use of anti-TNF and immunomodulatory therapy in CD had no influence on the control of patients' depressive symptoms. These findings differ from those obtained by Horst et al., who, through the PHQ-9 scores, found that depressive symptoms significantly decreased in the first months of treatment with anti-TNF.^25^ However, in agreement with the results evidenced in this present study, a meta-analysis showed that depression, anxiety, psychosis or suicide is not associated with the use of biologics in patients with IBD.^46^ The authors of this study understand that research on this subject should be encouraged to clarify and establish whether the use of biologicals has any action in controlling depression in patients with IBD.

Tobacco use seems to have a strong influence on depression in CD patients, but not in UC.^47^ In the sample of the present study, there was no significant difference in the rate of depression between smokers and non-smokers, but numerically the rate of depression was higher in smokers (61.2% vs. 36.6%).

80% of patients with CD may need a surgical procedure throughout their lives and 70% may need a second intestinal resection,^48^ however, in this present study, most patients had already undergone surgery (65.8%) evidencing the high degree of severity of the disease. Previous surgeries were not considered a risk factor for MDD in this research. Nahon et al. also observed that surgeries previously performed in patients with CD were not associated with the risk of depression.^41^

The predominant anatomical distribution of the disease, in accordance with the Montreal scale, followed an ileocolonic disease course (48.4%) similar to that observed in patients at a reference IBD clinic in the city of São Paulo (47.9%)^29^ and other studies performed in different countries such as Holland, which found a proportion of 47%,^49^ Italy 41.9%^32^ and Colombia 52.5%.^50^ Regarding the phenotype of the disease, the most prevalent was the penetrating one (47%), with values much higher than those reported by Gomes et al.^29^ (18%) and other authors.^32^^,^^51^ The isolated involvement of the colon occurred in 34.3% of the patients, ileal (6.7%), and were similar to those found in other studies.^50^^,^^52^

The most prevalent surgery was anal fistulectomy (25.8%), approximately 50% of patients had moderate or severe disease and were patients with a long time of disease (13.2 years). It was also observed that MDD was more associated with the inflammatory phenotype (45.5%) than fibrostenosing (37.8%) and penetrating (42.2%), with a statistically significant difference (*p* < 0.001). Regardless of the other features of the patients, the fibrostenosing and penetrating behavior presented a statistically lower chance of severe depression than the inflammatory behavior, respectively, 92% and 97% (OR = 0.03; 95% CI 0.0–0.18) lower. This is possibly due to the higher concentration of circulating pro-inflammatory cytokines (TNF-alpha, IL-6) present in those patients with greater inflammatory activity, which could explain the higher rate of MDD in these patients in relation to the fibrostenosing and penetrating phenotypes.^53^

It was not possible to identify a relationship between anatomical location and depressive disorder. The depression rate of patients with disease in the GIT, ileum, colon, and anal regions were similar between groups (p > 0.005). These results partially agree with those recently found by Marafini et al., who carried out a study involving 136 patients with CD and psychiatric diagnosis based on semi-structured interviews and not on the use of questionnaires, where they observed that psychiatric disorders were not related to behavior or to its anatomical location.^32^

This present study observed that patients in remission have a lower risk of MDD than those in activity, but this risk is not non-existent. Therefore, even those in clinical remission deserve special attention from a team with expertise in psychology. The therapeutic objective of these patients with CD should not only prioritize the GIT but a complete, holistic approach, enforcing the concept of health defined by the WHO, which is “the perfect physical, mental and social well-being”.

As a limitation of this study, it is mentioned that although the PHQ-9 is an effective, useful and widely used instrument to assess psychiatric disorders in patients with IBD,^46^ it is unable to provide a definitive diagnosis of this psychopathology. A structured interview with a psychiatrist can provide a true rate of patients with depressive illness and more appropriate treatment.^54^

In addition, the nature of the study, as it was cross-sectional, did not allow for investigation over time. It also did not allow the assessment of the cause/effect relationship, although it provided useful information to stimulate new studies and contribute to the management of depression in IBD in health policy in the country.

## Conclusion

The present results support data showing an increased prevalence of MDD in individuals with CD. Additionally, it indicates that MDD in CD might be related to the activity of CD. Prospective studies are warranted to confirm the present results and to address whether MDD leads to CD activity, CD activity leads to MDD or both ways are existent.

### Authors' contributions

Facanali, CBG: was responsible for the study conceptualization, data curation, writing- original draft. Sobrado, CW: Was involved in the study methodology, project administration, supervision. Fraguas, R: drafting, methodology, writing - review & editing. Facanali, MR: formal analysis, visualization. Boarini, LR: investigation, visualization. Sobrado, LF: visualization, conceptualization. Cecconello, I: validation, supervision, writing review & editing.

All authors contributed to the critical revising and the final approval of the manuscript.

## Conflicts of interest

The authors declare no conflicts of interest.
